# Supplementary material for: Skeletal Lipocalin-2 Is Associated with Iron-Related Oxidative Stress in ob/ob Mice with Sarcopenia
Source: Antioxidants (Basel). 2021 May 11;10(5):758. doi: 10.3390/antiox10050758 (PMC8150392; doi:10.3390/antiox10050758)
Supplement: Supplementary file 1 [file antioxidants-10-00758-s001.zip › antioxidants-1185448-supplementary.pdf]

Skeletal Lipocalin-2 is Associated with Iron-Related Oxidative Stress in ob/ob Mice with Sarcopenia

Eun Bee Choi [1], Jae Hun Jeong [1], Hye Min Jang [1], Yu Jeong Ahn [1], Kyu Hyeon Kim [2], Hyeong Seok An [1], Jong Youl Lee [1], Eun Ae Jeong [1], Jaewoong Lee [1], Hyun Joo Shin [1], Kyung Eun Kim[1] and Gu Seob Roh [1,*]

[1] Department of Anatomy and Convergence Medical Science, Bio Anti-aging Medical Research Center, Institute of Health Sciences, College of Medicine, Gyeongsang National University, Jinju, Gyeongnam 52777, Republic of Korea; dmsql2274@naver.com (E.B.C.); wogns3764@gmail.com (J.H.J.); gpals759@naver.com (H.M.J.); ahnujung@naver.com (Y.J.A.); gudtjr5287@hanmail.net (H.S.A.); jyv7874v@naver.com (J.Y.L.); jeasky44@naver.com (E.A.J.); woongs1111@gmail.com (J.L.); k4900@hanmail.net (H.J.S.); kke-jws@hanmail.net (K.E.K.)

[2] Department of Medicine, College of Medicine, Gyeongsang National University, Jinju, Gyeongnam 52777, Republic of Korea; kyuhyeunkim@gmail.com (K.H.K.)

* Correspondence: anaroh@gnu.ac.kr (G.S.R.); Tel.: +82-55-772-8035; Fax: +82-55-772-8039

Supplementary Table S1. List of qRT-PCR primers.

Gene name	Primer sequences
Telomere (telg)	Forward 5′ ACA CTA AGG TTT GGG TTT GGG TTT GGG TTT GGG TTA GTG T 3'<br>Reverse 5′ TGT TAG GTA TCC CTA TCC CTA TCC CTA TCC CTA TCC CTA ACA 3'
36b4d	Forward 5′ ACT GGT CTA GGA CCC GAG AAG 3'<br>Reverse 5′ TCA ATG GTG CCT CTG GAG ATT 3'
TNF α	Forward 5′ CCA GAC CCT CAC ACT CAG ATC 3'<br>Reverse 5′ CAC TTG GTG GTT TGC TAC GAC 3'
IL-6	Forward 5′ AGT TGC CTT CTT GGG ACT GA 3'<br>Reverse 5′ TCC ACG ATT TCC CAG AGA AC 3'
Timp1	Forward 5′ CGG TGG GTG GAT GAG TAA TG 3'<br>Reverse 5′ GGC TGC ACA GTG GAG AAT AA 3'
Lrp5	Forward 5′ GGA TGG GCA AGA ACC TCT ATT 3'<br>Reverse 5′ GTC AAG GTC TCT CCA CAC AAG 3'
HGF	Forward 5′ GGA CCA TGT GAG GGA GAT TAT G 3'<br>Reverse 5′ ATA CCA GGA CGA TTT GGG ATG 3'
MMP3	Forward 5 GGA CCA GGG ATT AAT GGA GAT G 3'<br>Reverse 5′ TGA GCA GCA ACC AGG AAT AG 3'
LCN2	Forward 5′ GCCATGGAGAAGAACCTGAA 3'<br>Reverse 5′ TTTCCAGGAAGTCACAGAGATG 3'
24p3R	Forward 5 TTG CAG GAA CTG GAG AAT ACC 3'<br>Reverse 5′ GTT GGT GAA GCC CAG GAT AA 3'
Tnfrsf1a	Forward 5′ GCT AGG TCT TTG CCT TCT ATC C 3'<br>Reverse 5′ CTT TCC AGC CTT CTC CTC TTT 3'

Supplementary Table S2. List of primary antibodies

Antibody	*Company*	*Catalog No.*	*Dilution*	*Applications*	*Source*
Perilipin 2	abcam	ab52356	1:1,000, 1:200	WB, IF	Rabbit
FOXO1	Sigma	#2880	1:1,000, 1:200	WB, IF	Rabbit
MuRf1	Santa Cruz	sc-398608	1:1,000, 1:50	WB, IF	Mouse
IL-6	Santa Cruz	sc-57315	1:1,000	WB	Mouse
Nrf2	Santa Cruz	sc-365949	1:1,000	WB	Mouse
NF-κBp65	Santa Cruz	sc-8008	1:1,000	WB	Mouse
NQO1	abcam	ab34173	1:1,000	WB	Rabbit
HO-1	Stressgen	SPA-895	1:1,000	WB	Rabbit
iNOS	Chemicon	AB5384	1:1,000	WB	Rabbit
F4/80	Santa Cruz	sc-377009	1:1,000	IHC	Mouse
LCN2	R&D	AF3508	1:1,000, 1:200	WB, IF	Goat
24p3R	Millipore	ABC846	1:1,000, 1;50	WB, IF	Rabbit
DMT1	Santa Cruz	sc-166884	1:1,000	WB	Mouse
Transferrin receptor	abcam	ab84036	1:1,000	WB	Rabbit
Ferritin	abcam	ab75973	1:3,000, 1:200	WB, IHC	Rabbit
Ferroportin	abcam	ab78066	1:1,000, 1:200	WB, IF	Rabbit
Hepcidin	Santa Cruz	sc-100277	1:1,000, 1:150	WB, IF	Mouse
p84	abcam	ab487	1:3,000	WB	Mouse
GAPDH	Sigma	#97166	1:10,000	WB	Mouse

WB, western blot; IF, immunofluorescence; IHC, immunohistochemistry

Supplementary Table S3. Upregulated genes in the skeletal muscles of ob/ob mice (FDR < 0.001).

Gene Symbol	Gene Info	logFC	FDR
**Gzmb**	granzyme B	5.94883	-1.14366
**Ccl3**	chemokine (C-C motif) ligand 3	5.083154	3.28E-05
**Ccl5**	chemokine (C-C motif) ligand 5	3.640853	5.81E-06
**Serpina3g**	serine peptidase inhibitor, clade A, member 3G	2.861471	0.000494
**Hck**	hemopoietic cell kinase	2.683933	1.38E-05
**Timp1**	tissue inhibitor of metalloproteinase 1	3.290359	7.6E-05
**Hgf**	hepatocyte growth factor	3.269319	1.3E-06
**Cav1**	caveolin 1, caveolae protein	2.751305	5.03E-05
**Adipoq**	adiponectin, C1Q and collagen domain containing	3.292517	0.000345
**Anxa1**	annexin A1	3.091036	3.51E-07
**App**	amyloid beta (A4) precursor protein	2.890011	1.17E-07
**Thbs1**	thrombospondin 1	4.0075	2.15E-05
**Angptl4**	angiopoietin-like 4	3.79912	3.53E-07
**Csf1r**	colony stimulating factor 1 receptor	2.13228	1.12E-06
**Gnb1**	guanine nucleotide binding protein (G protein), beta 1	1.705021	4.71E-05
**Lrp1**	low density lipoprotein receptor-related protein 1	2.094279	3.94E-05
**Anxa5**	annexin A5	1.929491	0.000577
**Lrp5**	low density lipoprotein receptor-related protein 5	1.300714	0.000151
**Fcgr2b**	Fc receptor, IgG, low affinity IIb	1.940922	6.62E-07
**Itga5**	integrin alpha 5 (fibronectin receptor alpha)	1.413288	8.72E-05
**Hmox1**	heme oxygenase 1	2.750012	2.53E-08
**Pf4**	platelet factor 4	2.143487	2.39E-07
**Igfbp3**	insulin-like growth factor binding protein 3	2.457293	9.42E-08
**Tnfrsf1a**	tumor necrosis factor receptor superfamily, member 1a	1.92376	8.74E-05
**Id3**	inhibitor of DNA binding 3	1.69551	0.000219
**Cd44**	CD44 antigen	2.241298	5.23E-05

**Tnfrsf1b**	tumor necrosis factor receptor superfamily, member 1b	2.230025	1.77E-05
**Sfrp1**	secreted frizzled-related protein 1	1.467531	0.000209
**Hmgcr**	3-hydroxy-3-methylglutaryl-Coenzyme A reductase	1.352907	0.000277
**Tgfb1**	transforming growth factor, beta 1	1.922796	3.81E-05
**Casp8**	caspase 8	1.876036	0.000134
**Irf5**	interferon regulatory factor 5	1.549624	0.000958
**Mmp3**	matrix metallopeptidase 3	4.747169	7.34E-08
**Lcn2**	lipocalin 2	4.080692	1.15E-10
**Plaur**	plasminogen activator, urokinase receptor	3.258796	8.65E-09
**Casp1**	caspase 1	3.108692	1.32E-06
**Src**	Rous sarcoma oncogene	1.706963	1.52E-05

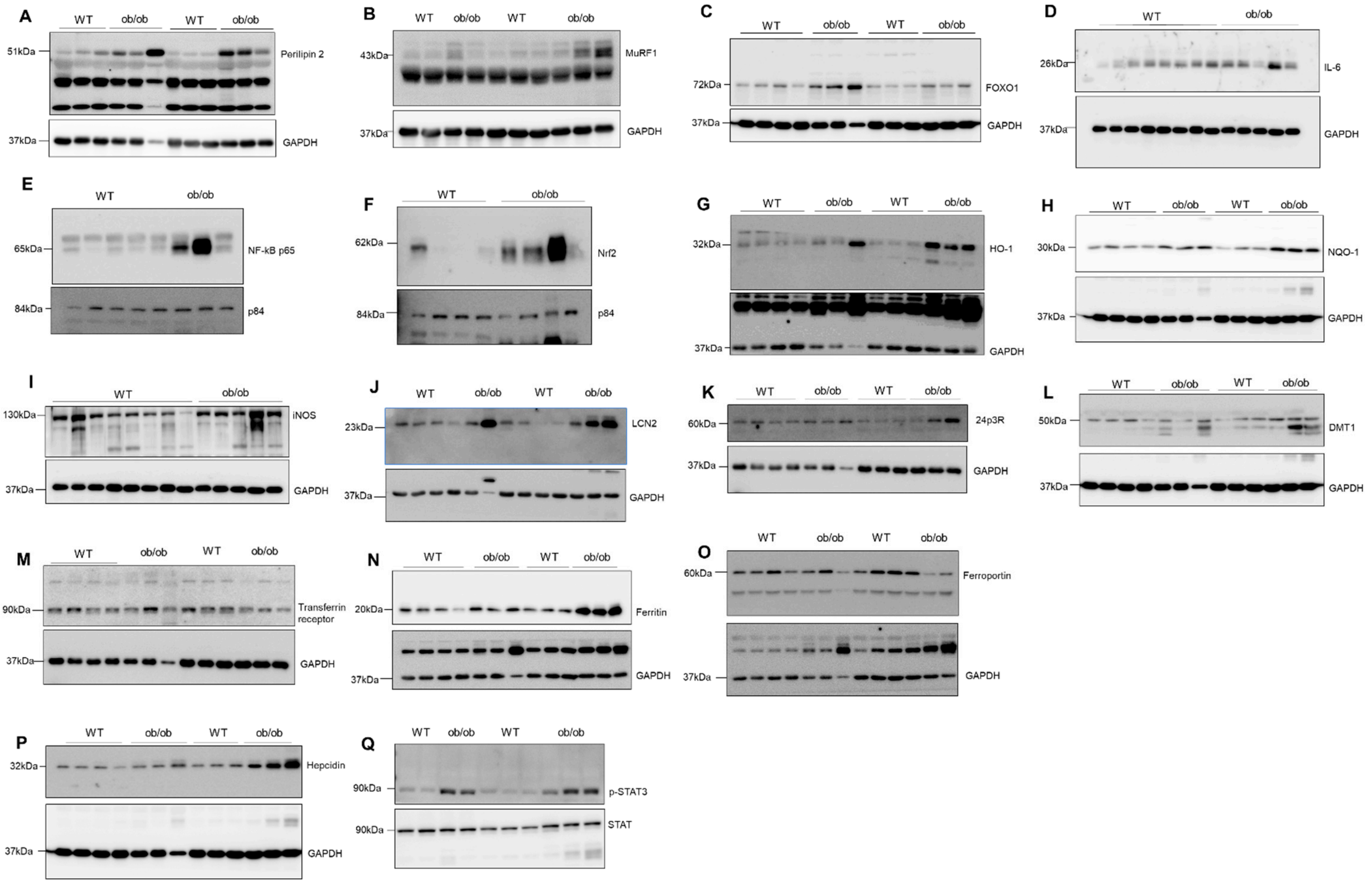

Supplemental Figure S1. Uncropped images from all western blots. (A-Q) figure 1D (A), figure 1H (B), figure 1I (C), figure 2D (D), figure 2F (E), figure 2G (F), figure 2H (G), figure 2I (H), figure 3C (I), figure 3D (J), figure 3D (K), figure 4C (L), figure 4D (M), figure 4E (N), figure 4G (O), figure 4H (P), and figure 4J (Q).

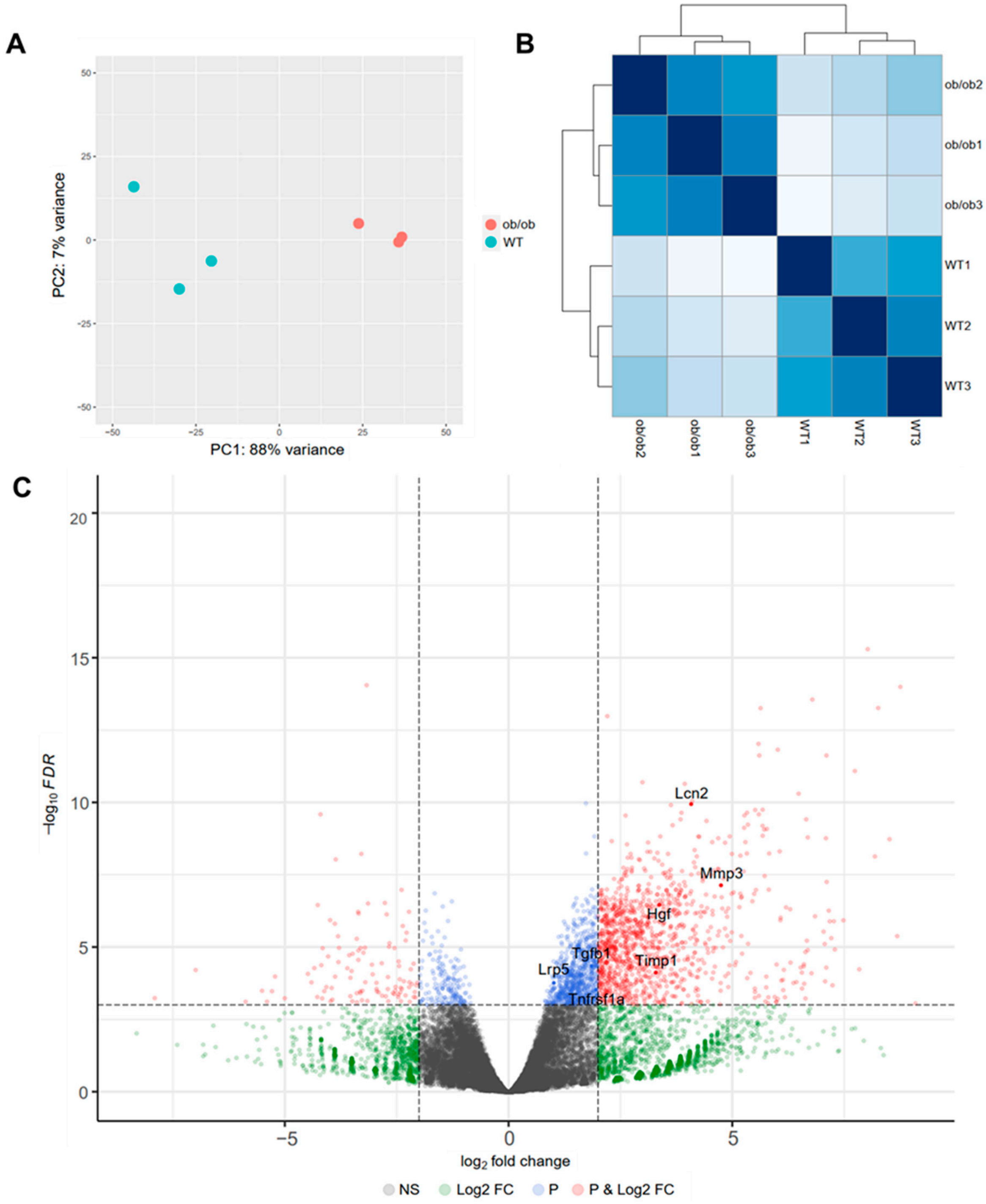

Supplemental Figure S2. Next-generation sequencing–based RNA-seq analysis of skeletal muscles of wild-type (WT) and ob/ob mice. (A) Principal component (PC) analysis, (B) gene correlation analysis, (C) Volcano-plot showing differentially expressed cell death–related genes as different-colored dots (*P* < 0.05, logFC > 2).

A

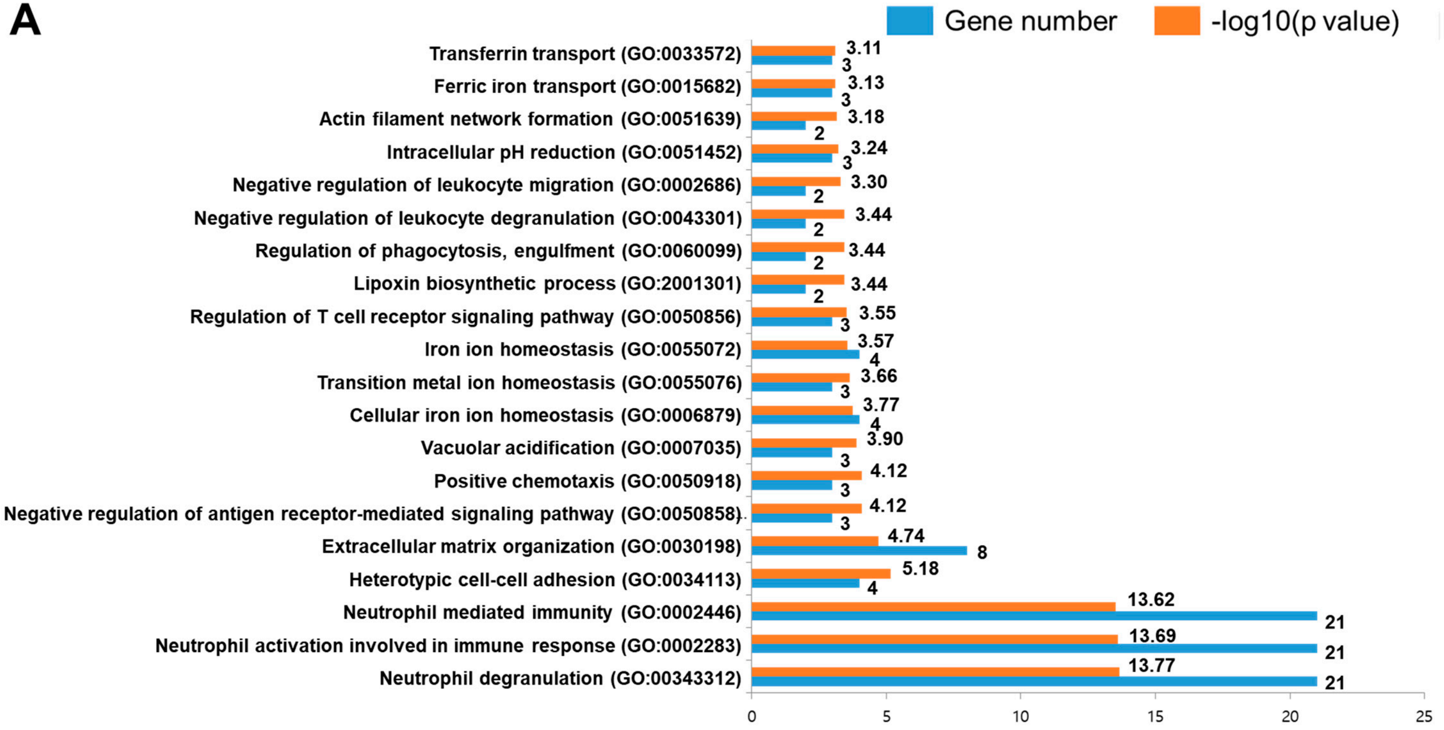

Gene number
-log10(p value)
Transferrin transport (GO:0033572)
3.11
3
Ferric iron transport (GO:0015682)
3.13
3
Actin filament network formation (GO:0051639)
3.18
2
Intracellular pH reduction (GO:0051452)
3.24
3
Negative regulation of leukocyte migration (GO:0002686)
3.30
2
Negative regulation of leukocyte degranulation (GO:0043301)
3.44
2
Regulation of phagocytosis, engulfment (GO:0060099)
3.44
2
Lipoxin biosynthetic process (GO:2001301)
3.44
2
Regulation of T cell receptor signaling pathway (GO:0050856)
3.55
3
Iron ion homeostasis (GO:0055072)
3.57
4
Transition metal ion homeostasis (GO:0055076)
3.66
3
Cellular iron ion homeostasis (GO:0006879)
3.77
4
Vacuolar acidification (GO:0007035)
3.90
3
Positive chemotaxis (GO:0050918)
4.12
3
Negative regulation of antigen receptor-mediated signaling pathway (GO:0050858)
4.12
3
Extracellular matrix organization (GO:0030198)
4.74
8
Heterotypic cell-cell adhesion (GO:0034113)
5.18
4
Neutrophil mediated immunity (GO:0002446)
13.62
21
Neutrophil activation involved in immune response (GO:0002283)
13.69
21
Neutrophil degranulation (GO:00343312)
13.77
21
0
5
10
15
20
25

B

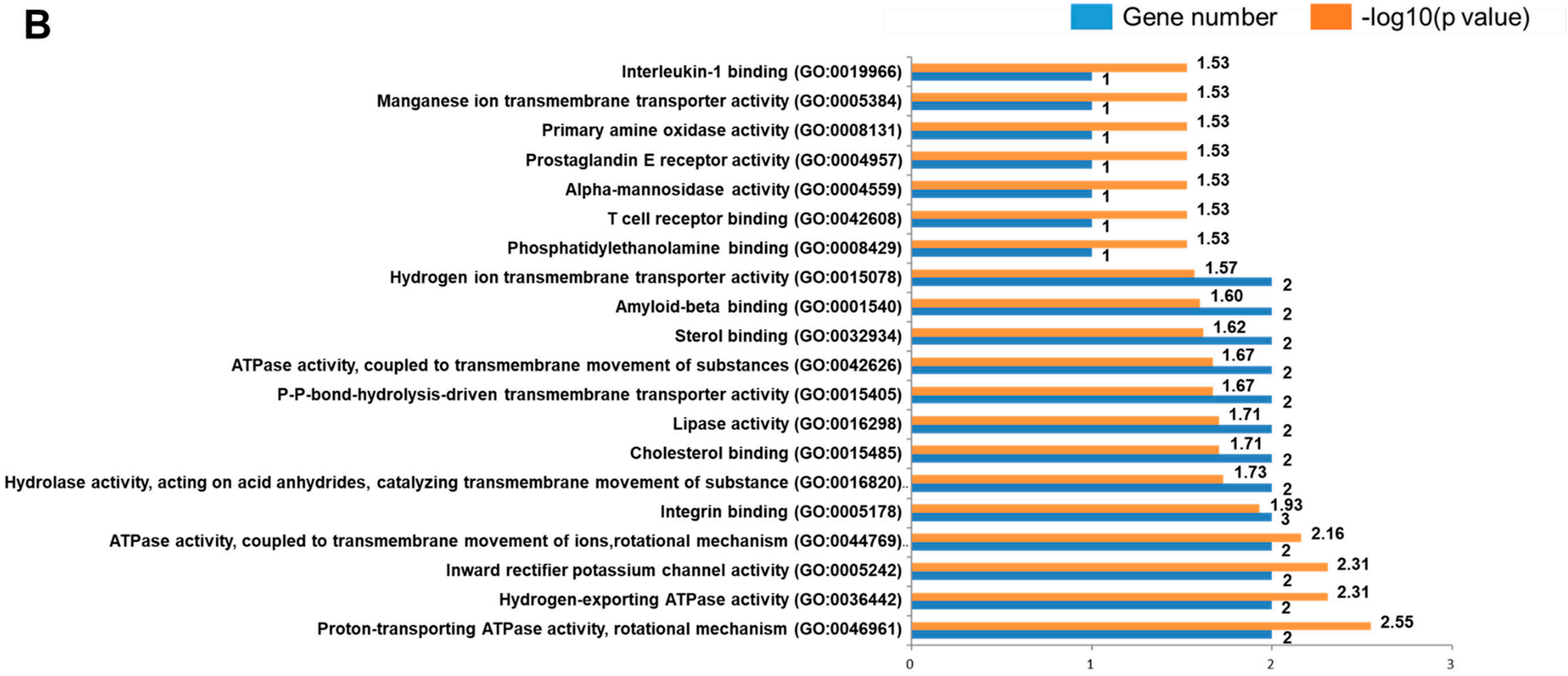

Gene number
-log10(p value)
Interleukin-1 binding (GO:0019966)
Manganese ion transmembrane transporter activity (GO:0005384)
Primary amine oxidase activity (GO:0008131)
Prostaglandin E receptor activity (GO:0004957)
Alpha-mannosidase activity (GO:0004559)
T cell receptor binding (GO:0042608)
Phosphatidylethanolamine binding (GO:0008429)
Hydrogen ion transmembrane transporter activity (GO:0015078)
Amyloid-beta binding (GO:0001540)
Sterol binding (GO:0032934)
ATPase activity, coupled to transmembrane movement of substances (GO:0042626)
P-P-bond-hydrolysis-driven transmembrane transporter activity (GO:0015405)
Lipase activity (GO:0016298)
Cholesterol binding (GO:0015485)
Hydrolase activity, acting on acid anhydrides, catalyzing transmembrane movement of substance (GO:0016820)
Integrin binding (GO:0005178)
ATPase activity, coupled to transmembrane movement of ions,rotational mechanism (GO:0044769)
Inward rectifier potassium channel activity (GO:0005242)
Hydrogen-exporting ATPase activity (GO:0036442)
Proton-transporting ATPase activity, rotational mechanism (GO:0046961)
1.53
1
1.57
1.60
1.62
1.67
1.71
1.73
1.93
3
2.16
2.31
2.55
2
0
1
2
3

C

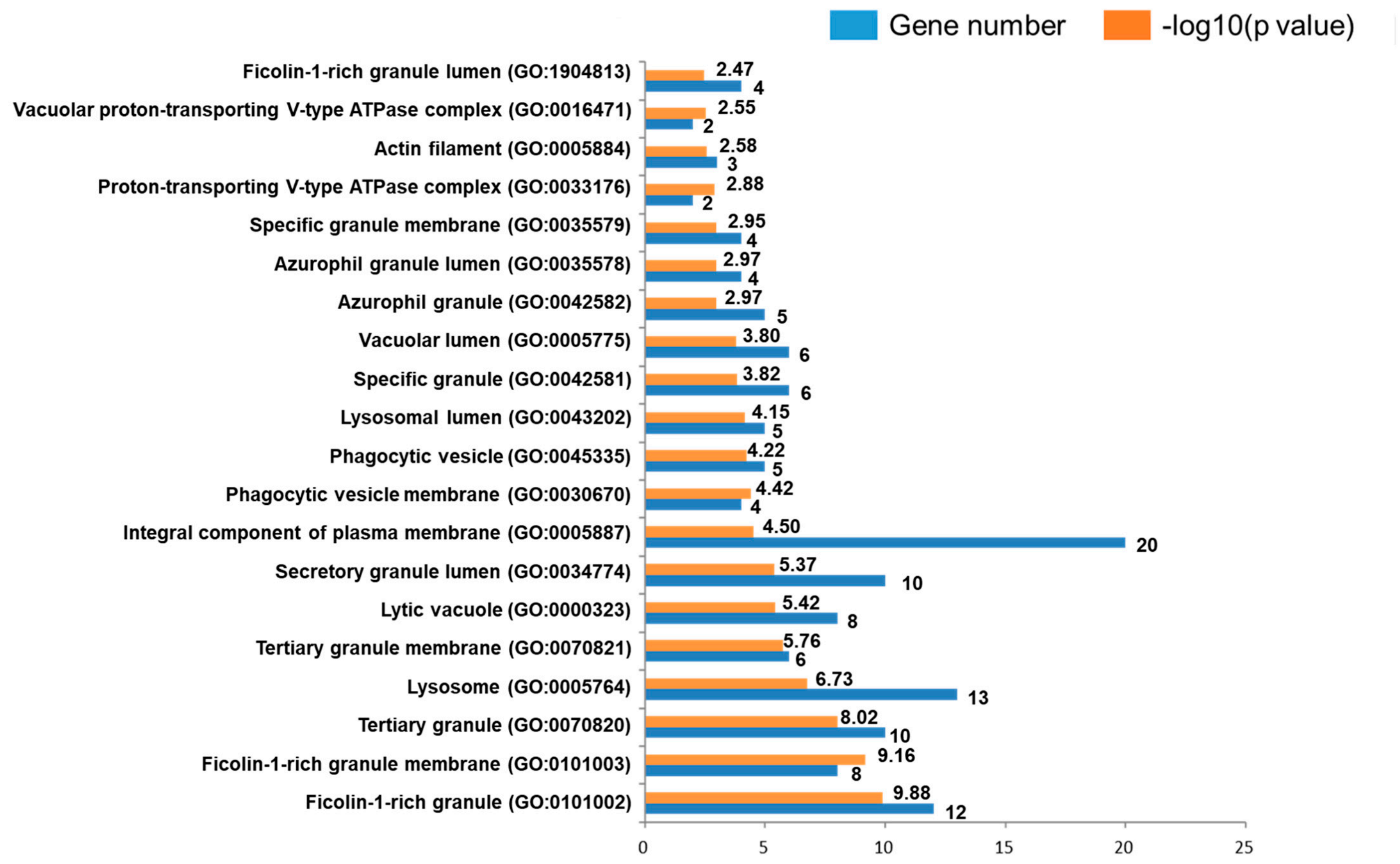

Supplemental Figure S3. Enriched Gene Ontology (GO) terms identified using enrichment analysis in skeletal muscles of wild-type and ob/ob mice. Only representative GO terms that include more than two sibling terms are displayed. (A–C) Biological process (A), Molecular function (B), Cellular component (C). Blue bars indicate the number of associated genes, and orange bars indicate −log (*P*-value) of the enrichment analysis.
